# Uterine Necrosis after Uterine Artery Embolization for Symptomatic Fibroids

**DOI:** 10.1155/2018/9621741

**Published:** 2018-05-28

**Authors:** Steve Kyende Mutiso, Felix Mwembi Oindi, Nigel Hacking, Timona Obura

**Affiliations:** ^1^Department of Obstetrics and Gynaecology, Aga Khan University, Nairobi, Kenya; ^2^University Hospital Southampton, Southampton, UK; ^3^Aga Khan University Hospital, Nairobi, Kenya

## Abstract

**Introduction:**

Uterine artery embolization (UAE) is a minimally invasive intervention that is used in the treatment of fibroids. UAE can lead to complications including postembolization syndrome, postprocedure pain, infection, endometrial atrophy leading to secondary amenorrhea, and uterine necrosis. Uterine necrosis after UAE is very rare and hence poses a clinical dilemma for any clinician in its identification and management. We document a case of uterine necrosis after UAE and conduct a literature review on its causation, clinical features, and management principles.

**Case:**

A patient presented one month after UAE with abdominal pain and abdominal vaginal discharge. Her work-up revealed features of possible uterine necrosis with sepsis and she was scheduled for a laparotomy and a subtotal hysterectomy was performed. She was subsequently managed with broad spectrum antibiotic and recovered well.

**Conclusion:**

Uterine necrosis after UAE is a rare occurrence and we hope the documentation of this case will add to the body of knowledge around it. Theories that explain its occurrence include the use of small particles at embolization, the use of Contour-SE a spherical poly-vinyl alcohol, and lack of collateral supply to the uterus. Its symptoms may be nonspecific but unremitting abdominal pain is invariably present. Finally although conservative management may be successful at times, surgical management with hysterectomy will be required in some cases. The prognosis is good after diagnosis and surgical management.

## 1. Introduction

Uterine artery embolization (UAE) is a minimally invasive intervention that is used in the treatment of fibroids [1]. UAE has been used for reduction of fibroid symptoms especially menorrhagia and offers relief to women not keen on surgical intervention [2, 3]. More so, UAE has been shown to be effective in reduction of fibroid symptoms and is also cost effective when compared to surgical management [4, 5]. Although its cost effectiveness has been disputed recently when compared to surgical interventions for myomas, UAE still remains a viable option for treatment of symptoms of leiomyomas [6, 7]. UAE has contraindications including pregnancy and malignancy, with relative contraindications including existing fertility desires and large myomas that are more than 8–10 centimeters [8].

UAE has various complications associated with it that vary from minor to major [7, 9]. These include postembolization syndrome, postprocedure pain, infection, persistent PV (per vaginam) discharge, fibroid passage PV, endometrial atrophy leading to secondary amenorrhea, nontarget embolization, and uterine necrosis [6]. UAE has also been associated with altered reproductive outcomes due to its associated altered ovarian function and premature ovarian failure in some cases [4, 9]. The rates of these complications vary from around 5.7% for intraprocedural complications, 37.3% for minor complications, and around 5% for major complication within the first year after UAE [7]. These complications may be comparable in rate to the ones of surgical management of fibroids which has a complication rate of 6.3% for intraprocedural complications, 23% for minor complications, and around 7% for major complication within the first year after [6]. However, uterine necrosis after UAE remains one of the rarest complications of UAE with only about 19 cases documented from the advent of UAE [10].

Uterine necrosis after UAE poses a clinical dilemma for any clinician in its identification and management [10, 11]. The hypotheses of its pathophysiology include the use of very small particles in UAE (<500 microns) and lack of arterial anastomoses to embolized regions among other theories [10]. The technical risk factors of necrosis also include unselective embolization and embolization till stasis is achieved [8, 10].

Uterine necrosis after UAE has few cases that are documented in literature. Moreover, it offers a diagnostic and management dilemma to clinicians when it occurs hence outlining its clinical significance. We document a case of uterine necrosis after UA and conduct a literature review on its causation, clinical features, and management principles.

## 2. Case

A 56-year-old African woman presented with symptoms of severe abdominal pain and brownish foul smelling vaginal discharge that had lasted for one month after UAE. She had associated symptoms of nausea and persistent vomiting but reported no fever or any other flu like symptoms. She had an associated nonproductive cough but no other respiratory symptoms.

She had a UAE done a month prior due to symptomatic uterine fibroids with her symptoms being menorrhagia and a feeling of an abdominal mass for a duration of one year. Her Pre-UAE MRi had shown multiple enhancing uterine fibroids numbering around 15 with the largest being around 6.5 centimeters. The UAE had been done with* Spongostan* Gelfoam slurry and she was discharged home after an overnight stay. Her discharge meds after UAE included antibiotics (cefuroxime and clindamycin for a week), analgesics (diclofenac and paracetamol), and metoclopramide as an antiemetic. She had been seen 2 weeks after the UAE with no major complaints with abdominal pain being minimal. This pain worsened afterwards and was associated with a fever that resolved after an antibiotic course (clindamycin and metronidazole).

Her known comorbidities were diabetes mellitus for a duration of 3 years and hypertension for a duration of 5 years for which she was being treated with lorsartan 50 milligrams once daily. She had not had any prior surgeries and had no other significant history in her past.

On examination she was in good general condition and was oriented in time and place. Her vitals revealed a tachycardia with a pulse of 107 beats per minute with a normal blood pressure of 104/67 millimeters of mercury and a temperature of 36.2 degrees Celsius. Abdominal examination revealed lower abdomen tenderness with a palpable pelvic mass at 14 weeks. The rest of her systems were normal in signs.

Investigations done included a full blood count which revealed a low hemoglobin level at 9.4 grams per deciliter with a normal white cell and platelet count. Her urea, creatinine, and electrolytes were all within normal limits. She had a chest X-ray done that revealed interstitial edema with borderline cardiomegaly and a subsequent computed tomography pulmonary angiogram revealed no evidence of pulmonary embolism.

**Figure 1:**
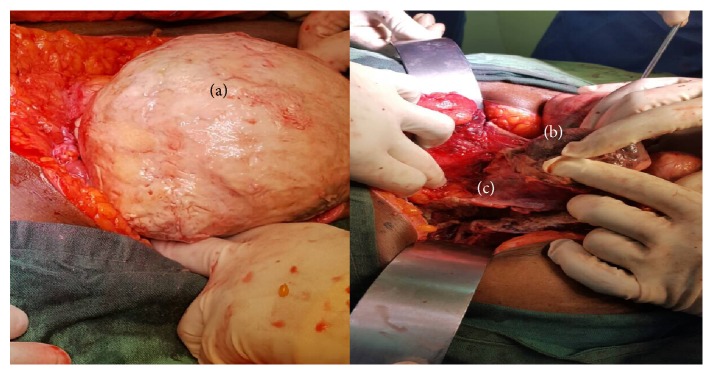
Necrosed uterus (a) and matted Necrosed Myomas (b). Serosal adhesions to small and large bowel (c).

**Figure 2:**
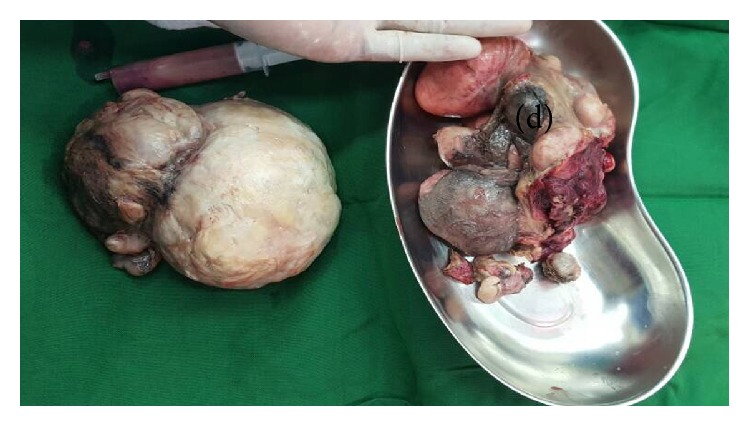
Matted Necrosed Myomas and the uterus (d) after extraction from the subtotal hysterectomy.

In view of her worsening condition with first-line treatment and nonremitting abdominal pain, it was decided that a surgical intervention would be appropriate and since she was postmenopausal, a total hysterectomy was advised. She was subsequently scheduled for the surgery which was done on day 2 of admission. Intraoperatively we found a Necrosed uterus with a thin serosal separation that was adhered to small and large bowel (Figure 1). Necrosed Myomas were found as a distinct single matted mass of around 12 myomas (Figure 2). Multiple small pelvic abscesses were also found. A general surgery team was invited to assist in her surgery at this point. The surgery done entailed drainage of the pelvic abscesses, separation of the adhered uterine wall serosa from small bowel, and a subtotal hysterectomy. Peritoneal lavage was done with around 10 liters of 0.9% sodium chloride solution. Bilateral Jackson Pratt drains were left in situ in both pelvic gutters to drain the pelvis.

Postoperatively, she was started on intravenous antibiotics (piperacillin, tazobactam, and clindamycin) and analgesia and thromboprophylaxis with enoxaparin. She required a relook laparotomy on day 4 after surgery due to wound sepsis in which she had abdominal washouts and fascial closure. Her skin incision was left open for subsequent dressing and secondary wound closure. Her wound swab had a positive culture of* Klebsiella pneumoniae* sensitive to Meropenem which she was started on postoperatively. She recovered as an inpatient for a further 10 days and was subsequently discharged after 16 days of admission.

At her follow-up at the clinic 2 weeks afterwards, she reported marked improvement with no major concerns. She underwent subsequent wound dressing and the wound healed well and did not require secondary closure. She was discharged from follow-up care about 3 months after the surgery.

## 3. Discussion

Uterine necrosis after UAE is a rare complication. The outlined case report documents it and offers insight towards salient feature of its management.

The exact incidence of uterine necrosis after UAE is difficult to ascertain with only a handful of cases reported in literature [6, 10]. The exact pathophysiology of its causation is not well known although there are a few theories that try to explain why it occurs [10]. The first theory postulates that use of fine particles (<500 micrometers) may predispose to post-UAE necrosis since they may embolize even the collateral supply to the uterus provided by the cervicovaginal and utero-ovarian vessels [12]. The other theory postulates that use of a specific spherical poly-vinyl alcohol agent (PVA) as an embolizing agent may also predispose to uterine necrosis [13]. Moreover, patients who do not have good collateral anastomosis between uterine and ovarian arteries at embolization may also be at increased risk of necrosis and hence embolization is avoided if one sees collaterals at the catheter position [14]. Additionally, nonselective embolization during UAE and embolization till stasis also predispose to uterine necrosis and so selective embolization is also a key factor in trying to prevent necrosis [8, 10]. Lastly other theories that exist include the lack of antibiotic prophylaxis after UAE and the existence of sepsis [15]. The current patient had gelatin used for her embolization and the size of the particles was larger than 500 micrometers; she also had collateral anastomosis to the uterus and we used antibiotic prophylaxis after UAE and hence none of the postulated theories seem to explain the occurrence of uterine necrosis in her.

The symptoms of uterine necrosis may be nonspecific. Most commonly the reported clinical picture includes abdominal pain, fever, leucorrhea, and menorrhagia at times [10]. Patients may also present with symptoms of sepsis if concurrent infection is present [7]. The current patient had most of the above symptoms with abdominal pain and abnormal discharge; she was however not septic at presentation. Other symptoms may occur with nontarget embolization leading to concurrent necrosis of adjacent organs such as the bladder, adnexa, vagina, or even the labia which have all been reported in literature [14, 16]. The current patient had no other concurrent organs involved and hence did not have target symptoms of these organs. Clinical acumen and ultrasound may be sufficient in the diagnosis of uterine necrosis after UAE although additional imaging such as a computed tomography scan and magnetic resonance imaging may help in its diagnosis [10, 16]. The current patient did not have any preoperative scans and a clinical diagnosis was sufficient.

The treatment of uterine necrosis usually involves either removal of the Necrosed portion of the uterus and myomas or a hysterectomy [15, 17]. Conservative management has also been described in literature as an option for a handful of patients [18]. The choice of the option of treatment largely depends upon the severity of symptoms of necrosis and associated complications [11, 15]. The current patient had severe abdominal pain and also had failed conservative management with antibiotics and oral analgesics, hence the decision for surgery and hysterectomy. Additional measures that seem to aid in the good outcome of such patients are the treatment of concurrent infection with broad spectrum antibiotic and multidisciplinary management in cases of adjacent organ involvement [15, 16]. The current patient had concurrent antibiotics after the surgery due to sepsis and also had a surgical team involved in her treatment and subsequently seemed to recover well.

In conclusion, uterine necrosis after UAE is a rare occurrence and we hope the documentation of this case will add to the body of knowledge around it. Theories that explain its occurrence include the use of small particles at embolization, the use of Contour-SE a spherical poly-vinyl alcohol, and lack of collateral supply to the uterus. Its symptoms may be nonspecific but unremitting abdominal pain is invariably present. Finally although conservative management may be successful at times, surgical management with hysterectomy will be required in some cases. The prognosis is good after diagnosis and surgical management.
